# Down-regulation of SLC14A1 in prostate cancer activates CDK1/CCNB1 and mTOR pathways and promotes tumor progression

**DOI:** 10.1038/s41598-024-66020-1

**Published:** 2024-06-28

**Authors:** Jianbin Ma, Kaihua Xue, Yifan Jiang, Xinyang Wang, Dalin He, Peng Guo

**Affiliations:** 1 Department of Urology, Qujiang Hospital, Northwest Corner of Huang Qutou Road Number Two and Changming Road, Xi’an, 710061 Shaanxi China; 2 Department of Urology, The First Affiliated Hospital of Xi’an Jiaotong University, 277 Yan-Ta West Road, Xi’an, 710061 Shaanxi China

**Keywords:** Cancer, Cell biology, Molecular biology

## Abstract

Prostate cancer (PCa) is the most common cancer among men in the United States and the leading cause of cancer-related death. The Solute Carrier Family 14 Member 1 (SLC14A1) is a member of urea transporters which are important for the regulation of urine concentration. However, the physiological significance of SLC14A1 in PCa still remains unclear. In the present study, via bioinformatics analysis and experiments, we found that expression of SLC14A1 is significantly decreased in PCa progression, which could be attributed to hypermethylation on SLC14A1 promoter region. Moreover, its low expression and hypermethylation on SLC14A1 promoter are closely related to the poor prognosis of PCa patients. On the other hand, overexpression of SLC14A1 inhibited cell proliferation and metastasis while its overexpression also suppressed CDK1/CCNB1 pathway and mTOR/MMP-9 signaling pathway. Additionally, SLC14A1 expression is enriched in prostate basal-type cells. In summary, our study indicates that its low expression level and promoter hypermethylation of SLC14A1 may represent novel indicators for PCa progression and prognosis, and SLC14A1 could inhibit the progression of PCa.

## Introduction

Prostate cancer (PCa) is the most common cancer among men in the United States and the second leading cause of cancer-related death^1^. Although localized disease is associated with a good prognosis, the 5-year survival rate for patients with metastatic PCa has dropped dramatically to 30%^2^. For decades, androgen deprivation therapy (ADT) has been the first treatment choice for patients with PCa. However, many patients, who initially respond, develop resistance to ADT and eventually develop to metastatic castration resistant PCa (mCRPC). Several sequencing studies have identified various PCa drivers, such as loss of *TP53*, amplification of *c-MYC* and *MYCN*, and alterations in PI3K, WNT and/or DNA repair pathways were enriched in advanced PCa^3^. Nevertheless, the exact causes for the development of PCa are still unclear. Thus, there is an urgent need to develop novel diagnostic and therapeutic approaches to better understand the development of PCa and provide novel targets for treatment.

There are several mechanisms of gene downregulation in PCa, mainly including gene deletion and epigenetic modifications. As we known, frequent deletions, such as loss of *PTEN*, *TP53* and *RB1*, occur in PCa progression^4,5^, and PCa shows poor outcomes with loss of the potent tumor suppressors described above^6^. Some other genes including *APC*, *ATM*, *BRCA1/BRCA2*, *CHD1*, *ERF*, *KMT2A*, *SETD2* are deleted to varying degrees in the progression and development of PCa^4^. DNA methylation, one of the important epigenetic modifications, plays essential roles in tumor initiation and progression of PCa. It has been reported that the methylation status of specific genes could be considered as potential tumor biomarker for the early diagnosis and prognosis of patients with PCa^7^. Meanwhile, hypermethylation of CpG islands is an important mechanism for gene silencing and downregulation in PCa^8^. Most importantly, hypermethylation of DNA promoter is not only associated with poor outcomes, clinical stage or pathological grade of PCa patients, but also it contributes to the abilities of invasiveness and metastasis of prostate tumors^9^. In PCa, several genes with promoter methylation have been identified, such as *GSTP1*, *CDKN2A* and *APC*^7^. However, we still need to further explore the significance of DNA methylation in PCa progression.

There are three epithelial cell types in the prostate gland, including luminal cells, which express CK8, CK18 and high levels of AR, basal cells, which express CK14 (encoded by *KRT14*), p63 and CK5 (encoded by *KRT5*), and rare neuroendocrine cells^10,11^. So far, in both luminal and basal layers, stem cell populations of prostate gland have been identified. It is worth noting that subpopulations of basal cells, which are isolated using cell-surface markers, exhibit self-renewal and multipotency^10^, indicating prostatic basal cells may be an important subcellular population for recurrence of PCa after treatment; therefore, it is crucial to explore potential surface marker of basal cells and identify subpopulations of basal cells.

The Solute Carrier Family 14 Member 1 (SLC14A1), also named UTB, is a member of urea transporters which are important for the regulation of urine concentration^12^. The human *SLC14A1* gene encoding a B-type urea transporter protein was localized on chromosome 18q12.3, adjacent to another urea transporter protein *SLC14A2* locus^13^. Two independent large-scale genome-wide association studies (GWAS) of uroepithelial bladder cancer found that mutations in the SLC14A1 gene were associated with human bladder carcinogenesis^14^. A recent meta-analysis of genome-wide association study of UBUCs found that the variant SLC14A1-rs10775480 at intron 6 was highly associated with susceptibility to bladder cancer, suggesting that SLC14A1 may play a causal or regulatory role^15^. Recently, it has been reported that SLC14A1 gene is a novel tumor suppressor in Urothelial carcinoma (UCs), and the study further demonstrated that SLC14A1 inhibited cell viability, proliferation, migration, invasion, tumor growth and metastasis^13^. Most recently, Ma et al.^16^ reported that interferon-dependent SLC14A1^+^ cancer-associated fibroblasts promote cancer stem cells via WNT5A in bladder cancer, elucidating the importance of SLC14A1 in the development of bladder cancer based on the tumor microenvironment. In PCa, it has reported that SLC14A1 is down-regulated in PCa and down-regulation of SLC14A1 promotes biochemical recurrence (BCR)^17^, and another research showed that castration affects SLC14A1 expression^18^. However, the mechanism of SLC14A1 down-regulation and the biological significance still remain unclear.

In the present study, via bioinformatics and public database analysis, we detected the expression of SLC14A1 and hypermethylation of SLC14A1 promoter in PCa, and analyzed their correlation with PCa progression and whether the expression of SLC14A1 was associated with promoter hypermethylation. Moreover, we overexpressed SLC14A1 in PCa cells and investigated whether SLC14A1 regulates cell proliferation in vitro and in vivo, and further identified the downstream pathways modulated by SLC14A1. In addition, to investigate whether SLC14A1 could be a specific marker in the prostate tissue, we analyzed single-cell transcriptomic sequencing data of adult prostate in the GRNdb database. Our study indicates that the expression level and promoter hypermethylation of SLC14A1 may represent novel indicators for PCa progression and prognosis, and SLC14A1 could inhibit the progression of PCa.

## Results

### Expression of SLC14A1 is significantly decreased in PCa progression, and its downregulation is associated with shorter survival of patients

**Figure 1:**
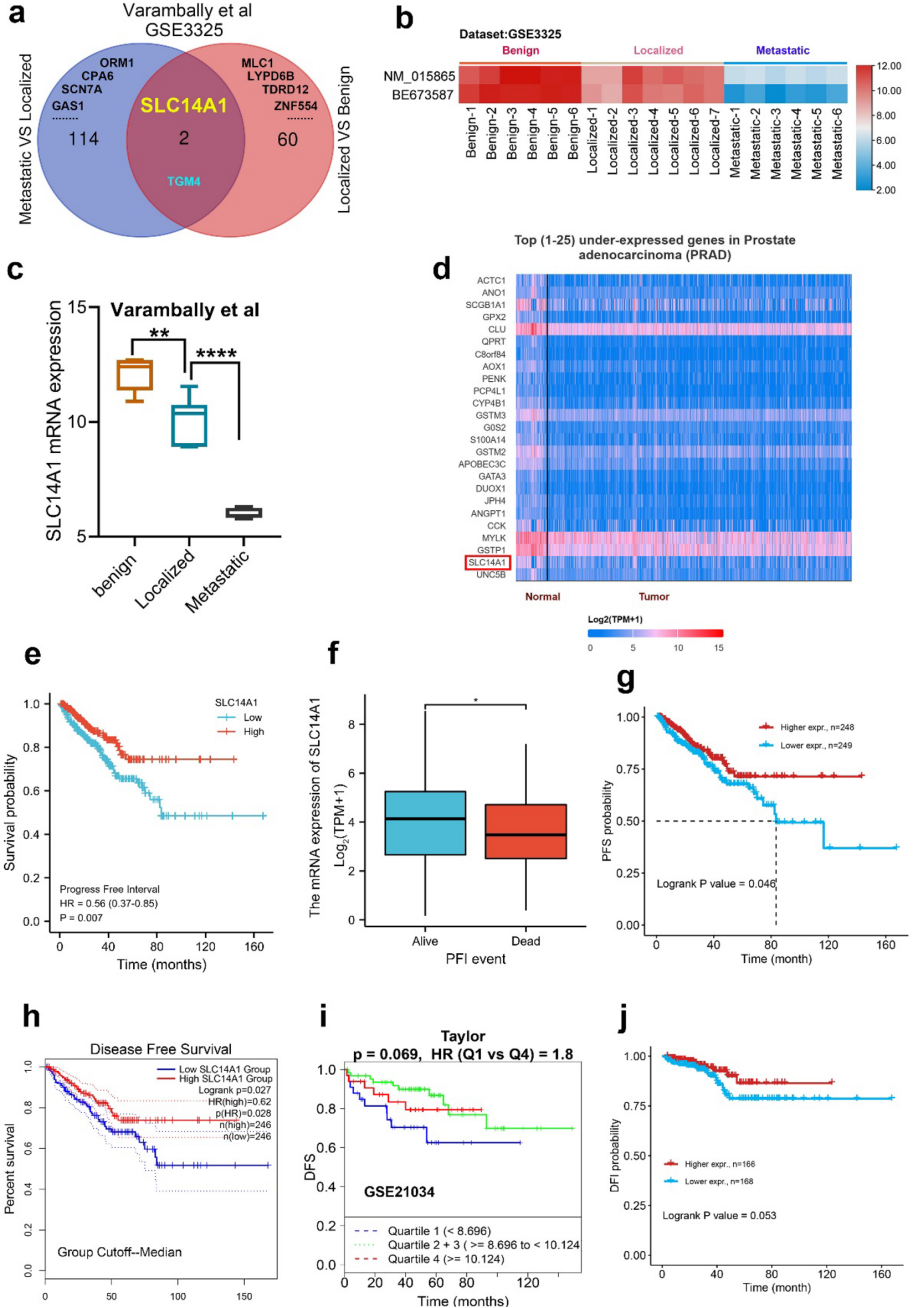
Expression of SLC14A1 is significantly decreased in PCa progression, and its downregulation is associated with shorter survival of patients. (**a**) Venn diagram indicates SLC14A1 downregulates in localized tissues (Localized vs. Normal) and metastatic tissues (Metastatic vs. Localized) as analysis from GSE3325 dataset. (**b**) A heatmap shows expression of SLC14A1 different transcripts in PCa progression through GSE3325 dataset. (**c**) SLC14A1 mRNA expression in PCa progression was analyzed from varambally et al. (GSE3325). (**d**) Top 25 under-expressed genes prostate adenocarcinoma (PRAD). (**e**–**g**) Analysis of the association between SLC14A1 mRNA expression and survival rate (progression-free interval or progression-free survival) of PCa patients through R language and GSCA website (http://bioinfo.life.hust.edu.cn/GSCA/#/). (**h**–**j**) Analysis of the association between SLC14A1 expression and survival rate (disease-free survival or disease-free interval) of PCa patients by applying TCGA, GSE21034 and GSCA database.

To identify valuable markers for PCa progression, we analyzed GSE3325 dataset (Varambally et al.) consisting of 6 normal prostate tissues, 7 PCa tissues and 6 metastatic PCa tissues. Through GEO2R online tool, we extracted 116 and 62 DEGs from metastatic tissues (metastatic vs. localized, log FC < − 4) and localized tissues (localized vs. normal, log FC < − 2) respectively. Then based on Venn diagram software, we identified SLC14A1 as the common DEG (Fig. 1a). For visualization, we extracted the transcripts expression data of SLC14A1 in the GSE3325 dataset and further analysis found that SLC14A1 was significantly downregulated in PCa progression (Fig. 1b,c), meanwhile, by the analysis from TCGA database, we found that downregulation of SLC14A1 ranked the 24th among the top 25 low-expressed genes in PCa (Fig. 1d), indicating that the downregulation of SLC14A1 plays an important role in PCa progression.

We further analyzed the correlation between SLC14A1 expression and survival of PCa patients. Firstly, we applied R language analysis and found that low expression of SLC14A1 leads to a poor progression-free interval (PFI) rate (Fig. 1e), moreover, according to TCGA database, SLC14A1 expression level in cancer tissues of dead PCa patients was significantly lower than that of living patients based on progression-free interval event (Fig. 1f). By further analyzing GSCA database we found that low expression of SLC14A1 leads to a poor progression-free survival (PFS) rate (Fig. 1g). Next, by analyzing GEPIA website and GSE21034 dataset (Taylor et al.), we found a low SLC14A1 expression level leads to a poor disease-free survival (DFS) rate in PCa patients (Fig. 1h,i). Similarly, we found low expression of SLC14A1 leads to a poor disease-free interval (DFI) rate by mining GSCA online database (Fig. 1j).

Notably, the results of the univariate logistic regression analyses showed that there were certain clinicopathological differences between the groups with high and low expression of SLC14A1, including T stage, N stage, Gleason score and Residual tumor.

(Table 1). In short, SLC14A1 is obviously lowly expressed in the occurrence and development of PCa, and its low expression is significantly associated with poor prognosis in PCa patients.

**Table 1:** Associations of SLC14A1 expression with clinicopathological characteristics of PCa patients.

Characteristics	Total (N)	Odds ratio (OR)	*p* value
T stage (T3&T4 vs. T2)	488	0.638 (0.441–0.921)	**0.017**
N stage (N1 vs. N0)	422	0.504 (0.300–0.833)	**0.008**
M stage (M1 vs. M0)	456	0.511 (0.024–5.373)	0.585
Gleason score (9&10 vs. 6&7)	432	0.408 (0.268–0.616)	** < 0.001**
Age (> 60 vs. <=60)	495	0.855 (0.600–1.219)	0.388
PSA (ng/mL) (>=4 vs. < 4)	438	0.552 (0.239–1.215)	0.148
Residual tumor (R1&R2 vs. R0)	465	0.630 (0.425–0.931)	**0.021**

Significant values are in [bold].

## SLC14A1 is significantly downregulated in PCa at both the mRNA and protein levels

**Figure 2:**
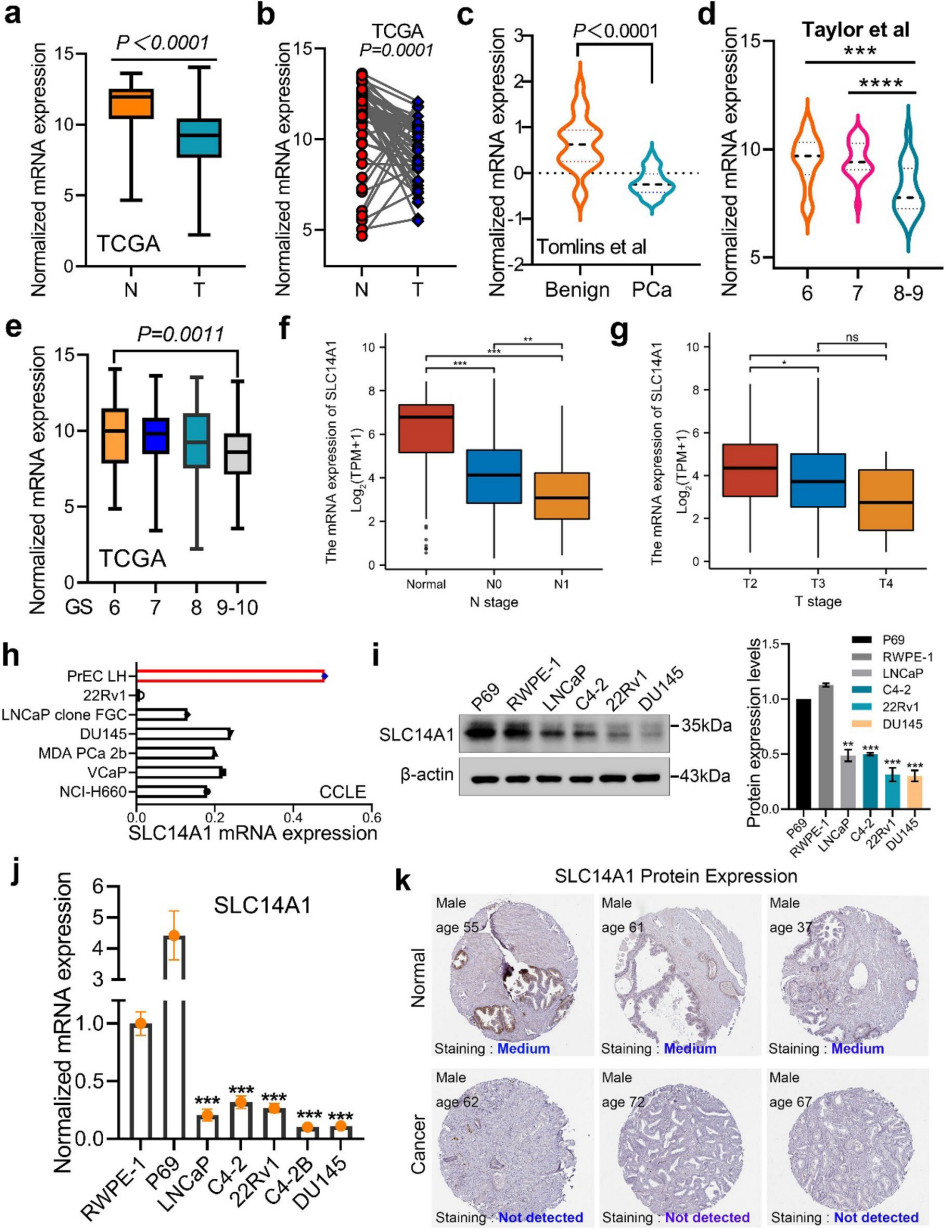
SLC14A1 is significantly downregulated in PCa at both the mRNA and protein levels. (**a**) SLC14A1 mRNA expression in PCa tissues and normal adjacent tissues based on unpaired or (**b**) paired from TCGA database. (**c**) Analysis of SLC14A1 mRNA expression in adjacent tissues and PCa tissues from GSE6099 dataset (Tomlins et al.). (**d**,**e**) Analysis of mRNA expression of SLC14A1 in PCa tissues with different Gleason score from GSE21032 (Taylor et al.) dataset and TCGA database. (**f**,**g**) Analysis of SLC14A1 mRNA expression in PCa tissues with different N stage or T stage. (**h**) Relative mRNA expression of SLC14A1 in cell lines of PCa analyzed from CCLE online database (https://sites.broadinstitute.org/ccle/). (**i**) Protein expression levels of SLC14A1 in PCa cell lines detected by western blotting, β-actin was used as internal loading control (n = 3, mean ± SD). (**j**) Analysis of mRNA expression levels of SLC14A1 in PCa cell lines detected by Real-time qPCR, 18S was used as internal loading control (n = 3, mean ± SD). (**k**) Representative pictures of SLC14A1 protein expression in PCa tissues and adjacent tissues detected by IHC from THPA online database (https://www.proteinatlas.org/). *p < 0.05; **p < 0.01; ***p < 0.001; ****p < 0.0001; ns, not significant.

To investigate in detail the expression of SLC14A1 in PCa, we analyzed public data [TCGA database and GSE6099 dataset (Tomlins et al.)] and observed that SLC14A1 mRNA is significantly downregulated in PCa tissues compared to normal adjacent tissues (NAT) or normal prostate tissues whether paired or unpaired (Fig. 2a–c). Moreover, by analyzing GSE21032 (Taylor et al.) dataset, we observed that SLC14A1 mRNA is downregulated dramatically in high Gleason score 8–9, a crucial indicator to evaluate PCa progression, compared with low Gleason score 6 and 7 PCa tissues (Fig. 2d). We further demonstrated that SLC14A1 is downregulated in high Gleason score tissues via mining TCGA data (Fig. 2e). Next, we explored the expression of SLC14A1 in N stage and T stage of PCa progression based on TCGA database. Apparently, the expression of SLC14A1 was downregulated with the development and progression of N stage or T stage, in other words, SLC14A1 is significantly down-regulated in lymph node metastases or high T-stage tissues (Fig. 2f,g).

To further examine the expression pattern of SLC14A1 in PCa cell lines, we firstly analyzed CCLE database and observed that expression of SLC14A1 mRNA is decreased in PCa cell lines compared to normal prostate cell PrEC LH (Fig. 2h). Next, we found SLC14A1 expression is downregulated in the PCa cell lines (LNCaP, C4-2, 22Rv1and DU145) when compared with RWPE-1 and P69 cells at both the mRNA and protein level detected by qRT-PCR and western blotting assays (Fig. 2i–j). Consistent with the SLC14A1 protein expression pattern in cell lines, we further mined the THPA database containing IHC data and found that protein expression of SLC14A1 is not detectable in PCa tissues compared with medium expression in normal prostate tissues (Fig. 2k). Taken together, these results demonstrate that SLC14A1 is significantly downregulated in PCa, further indicating SLC14A1 could be viewed as a tumor suppressor.

### Hypermethylation of SLC14A1 promoter contributes to its significant downregulation in PCa

**Figure 3:**
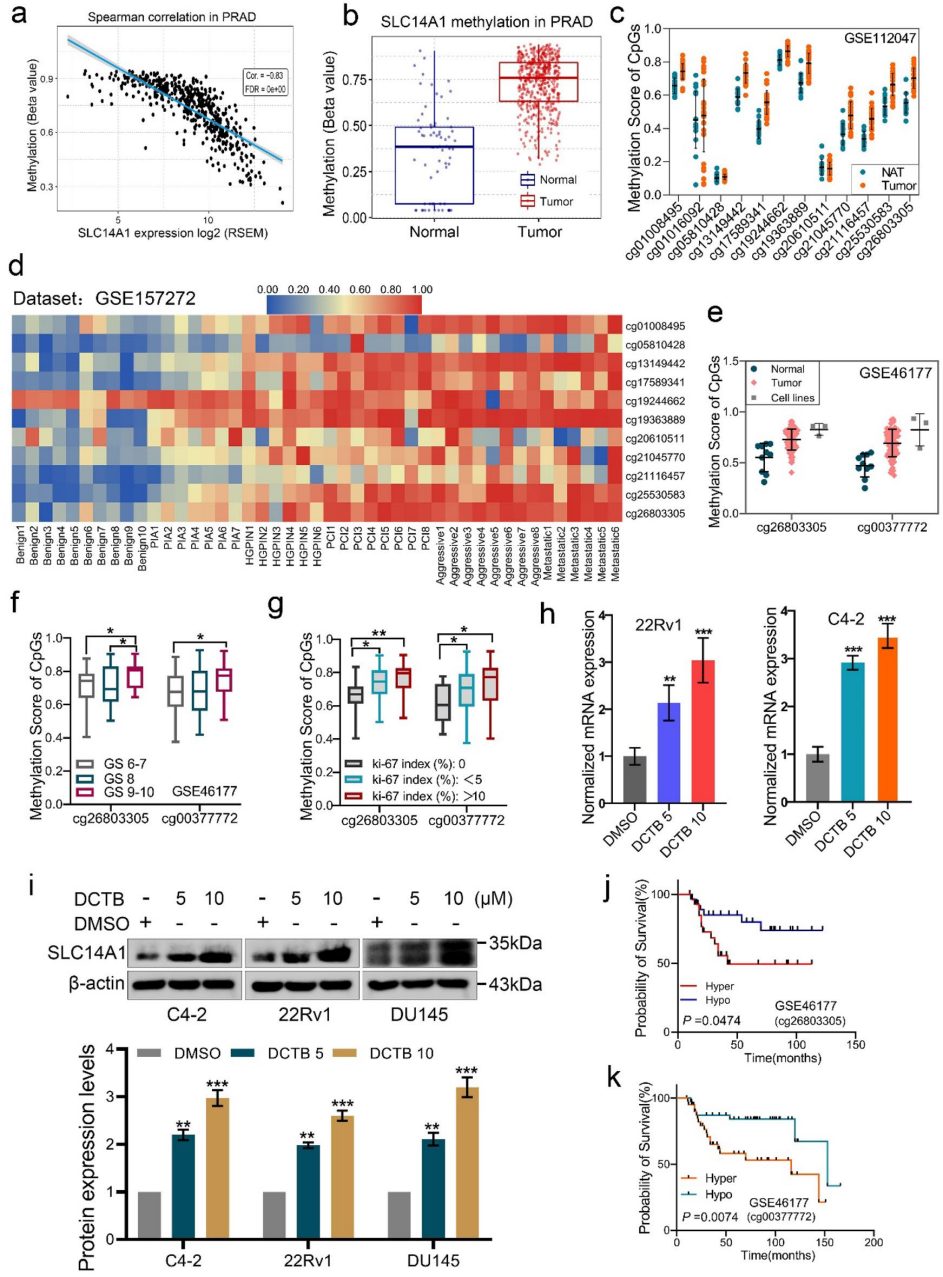
Hypermethylation of SLC14A1 promoter contributes to its significant downregulation in PCa. (**a**) The spearman correlation between methylation and SLC14A1 expression by analyzing the GSCA database. (**b**) Methylation levels of SLC14A1 promoter in adjacent tissues and PCa tissues analyzed from GSCA database. (**c**) Methylation levels of different CpG islands of the SLC14A1 promoter in PCa tissues and adjacent tissues by the analysis from GSE112047 dataset. (**d**) Heatmap of methylation levels of different CpG islands of the SLC14A1 promoter in the six different stages of PCa progression from GSE157272 dataset. Benign, benign prostate tissue; PIA, proliferative inflammatory atrophy tissue; HGPIN, high grade prostatic intra-epithelial neoplasia tissue; PCI, indolent prostate cancer tissue; Aggressive, aggressive prostate cancer tissue; Metastatic, metastatic prostate cancer tissue. (**e**) Methylation score of cg26803305 and cg00377772 in PCa tissues, cancer cells and NAT by the analysis from GSE46177 dataset. (**f**,**g**) Methylation score of cg26803305 and cg00377772 methylation sites detected in PCa tissues with different gleason scores or ki-67 index by the analysis from GSE46177 dataset. (**h**,**i**) The protein and mRNA levels of SLC14A1 in PCa cell lines treated with different concentrations of decitabine detected by qRT-PCR and western blotting assays (n = 3, mean ± SD). (**j**,**k**) Analysis of the association between the methylation level of cg26803305 or cg00377772 of SLC14A1 promoter and overall survival rate of PCa patients by analyzed from GSE46177 dataset. *p < 0.05; **p < 0.01. DCTB, decitabine. Hyper, hypermethylation; hypo, hypomethylation.

We have elucidated that SLC14A1 is significantly downregulated in PCa, however, the reason for its downregulation is still unknown. By analyzing TCGA data, we found nearly 23% of SLC14A1 gene are deleted in PCa, implying that DNA deletion may be one of reasons contributing to SLC14A1 down-regulation (Fig. S1a). However, no mutation was detected in the coding DNA sequence of the SLC14A1 gene (Fig. S1b). It has reported that SLC14A1 downregulation in bladder urothelial carcinoma is attributed to epigenetic silencing, i.e. hypermethylation^13^. Therefore, we further focused on the promoter methylation status of the SLC14A1 gene. Firstly, by analyzing the GSCA database (mainly based on TCGA), we found that hypermethylation of the SLC14A1 promoter was negatively correlated with SLC14A1 mRNA levels (Fig. 3a). Next, we observed methylation level of the SLC14A1 gene promoter was higher in PCa tissues when compared with normal prostate tissues based on GSCA database (Fig. 3b). As shown in Fig. 3c, higher methylation level was detected in the region of different CpG islands of the SLC14A1 gene in PCa tissues compared with normal adjacent tissues as evidenced by the analysis from GSE112047 dataset. To further explore the methylation status of the SLC14A1 in PCa progression, we analyzed the methylation dataset GSE157272. As shown in Fig. 3d, increasing levels of methylation of the SLC14A1 promoter were observed during the six stages of PCa progression. The heat-map clearly demonstrates that the significantly elevated methylation levels of CpG islands of SLC14A1 in PCa metastases. Similarly, based on the GSE46177 dataset, the methylation levels of cg26803305 and cg00377772 methylation sites were found to be significantly higher in PCa tissues versus cancer cells than in NAT (Fig. 3e).

As shown in Fig. 3f, the methylation levels of cg26803305 and cg00377772 methylation sites were increasing with higher Gleason scores (GS 9–10). It was also found that the high methylation levels of the two CpG islands were related to the high ki-67 index (Fig. 3g). The above results suggest that the methylation level of the promoter of SLC14A1 gene is increasing as PCa progresses. To further test and verify the above results, PCa cells were treated with different concentrations of decitabine (DCTB, an inhibitor of DNA methylation) for 6 days. The qRT-PCR and western blotting assays revealed that the expression of SLC14A1 at both the mRNA and protein level were increased by DCTB treatment (Fig. 3h,i). In conclusion, our results indicate that the downregulation of SLC14A1 in PCa is mostly due to its promoter hypermethylation. On the other hand, we analyzed methylation dataset GSE46177 and found that hypermethylation of cg26803305 and cg00377772 methylation sites in the promoter of SLC14A1 is related to poor overall survival of PCa patients (Fig. 3j,k).

DNA methyltransferases DNMT1, DNMT3A and DNMT3B play central roles in the development of cancers^19^. To further investigate the DNA methyltransferase that mediates methylation of the SLC14A1 gene, we firstly assessed the correlation of DNMT1, DNMT3A and DNMT3B with SLC14A1 by analyzing TCGA database, respectively. As shown in Fig. S2a–c, DNMT3A and DNMT3B were negatively correlated with SLC14A1, respectively. Next, we explored DNA transferases associated with SLC14A1 in GEO databases through the CANCERTOOL website. As shown in Fig. S2d–f, SLC14A1 was significantly negatively correlated with DNA methyltransferase DNMT3B as evidence by analysis from GSE35988 (Grasso et al.), GSE21032 (Taylor et al.) and GSE3325 (Varambally et al.). In summary, our results suggest that DNA methyltransferase DNMT3B may mediate methylation of the SLC14A1 promoter region and contribute to its low expression.

### SLC14A1 overexpression suppresses cell growth and inhibits CDK1/CCNB1 pathway

**Figure 4:**
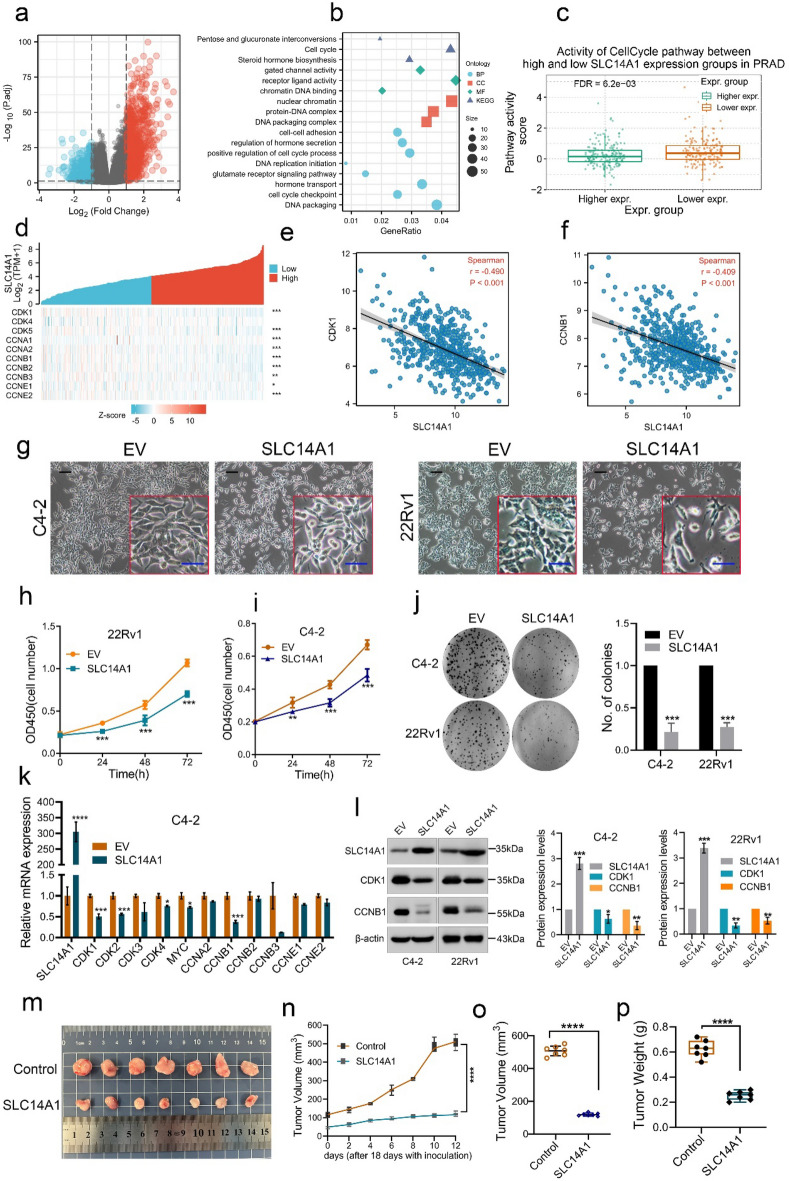
SLC14A1 downregulation promotes cell proliferation by regulating cell cycle pathway. (**a**) Volcano plot of DEGs via TCGA database. In details, red dots indicate the significantly downregulated DEGs; Blue dots indicate the up-regulated DEGs. DEGs, differentially expressed genes. (**b**) Go and KEGG analysis of downregulated DEGs by analyzed from R language. Go, Gene Ontology; BP, biological process; CC, Cellular Component; MF, Molecular Function. (**c**) Activity of cell cycle pathway between high and low SLC14A1 expression groups in PCa by the analysis from GSCA database. (**d**) Heatmap of the correlation between SLC14A1 mRNA expression and the cell cycle related genes. (**e**–**f**) Analysis of correlation between SLC14A1 and CDK1 or CCNB1 based on TCGA. (**g**) Representative microscopic images of the morphology of C4-2 and 22Rv1 cells after SLC14A1 overexpression. Scale bar = 100 μm. (**h**,**i**) MTT assay was performed to detect viability in C4-2 and 22Rv1 cells with SLC14A1 overexpression (n = 3, mean ± SD). (**j**) Colony formation assays were performed in C4-2 and 22Rv1 cells with SLC14A1 overexpression (n = 3, mean ± SD). (**k**) Effect of SLC14A1 overexpression on the mRNA expression of relative cell cycle related genes in C4-2 cells, as detected by RT-qPCR. 18S was used as an internal loading control (n = 3, mean ± SD). (**l**) Western blotting analysis of SLC14A1, CDK1 and CCNB1 in C4-2 and 22Rv1 treated as described above (n = 3, mean ± SD). (**m**) Representative images of tumors obtained from nude mice in the control vector group and SLC14A1-overexpressed group. (**n**–**p**) The volume and weight of tumors in the groups described above were measured and compared after the mice were sacrificed at the end of the experiment. β-actin was used as an internal loading control. *p < 0.05; **p < 0.01; ***p < 0.001; ****p < 0.0001.

A total of 1584 genes were differentially expressed between the groups with high and low expression levels of SLC14A1, including 1018 upregulated DEGs and 566 downregulated DEGs via TCGA database. (adjusted *p* value < 0.05, |Log2-FC|> 1) (Fig. 4a). Next, we performed GO and KEGG enrichment analysis of the down-regulated DEGs. As shown in Fig. 4b, GO enrichment analysis revealed that down-regulated DEGs were enriched in different GO terms, including cell cycle checkpoint, DNA replication initiation, positive regulation of cell cycle process, cell–cell adhesion and DNA packaging complex. Additionally, KEGG pathway analysis showed that down-regulated DEGs significantly enriched in pathways including cell cycle and steroid hormone biosynthesis. Further GSEA analysis of the downregulated DEGs revealed that the DEGs were mainly enriched in pathways including hallmark G2M checkpoint. It is worth noting that activity of cell cycle pathway in high-SLC14A1 expression is lower than in low-SLC14A1 expression group by the analysis from GSCA database (Fig. 4c). So that, the above single-gene bioinformatics analysis results suggest that SLC14A1 may affect PCa cell growth by regulating the cell cycle pathway.

To further explore the specific molecules involved in the cell cycle pathway, we found that among the large number of DEGs downregulated, the expression of some genes related to cell cycle was significantly negatively correlated with SLC14A1 expression, including CDK1, CKD4, CDK5, CCNA2, CCNB1, CCNB2, CCNB3, CCNE1 and CCNE2 (Fig. 4d), especially CDK1 and CCNB1. As shown in Fig. 4e,f, we observed the mRNA of SLC14A1 is negatively significantly correlated with CDK1 and CCNB1 by analyzing TCGA data. Notably, the most significant negative correlation was found between CDK1 and SLC14A1. The above results indicate that downregulation of SLC14A1 may promote cell proliferation and growth by up-regulating cell cycle-related genes. To verify the effect of SLC14A1 on PCa cell proliferation and growth, we used C4-2 and 22Rv1 cells as in vitro study models and further overexpressed SLC14A1 gene in these two cell lines. As shown in Fig. 4g, it is obvious that overexpression of SLC14A1 leads to a poor cell state from observation the cell morphology. The MTT assays showed that overexpression of SLC14A1 could significantly inhibit cell growth ability in vitro (Fig. 4h,i). Similarly, by performing colony formation assays in C4-2 and 22Rv1 cells, we found that overexpression of SLC14A1 obviously inhibited cell growth and colony-forming (Fig. 4j), moreover, as shown in Fig. 4m–p, SLC14A1 overexpression inhibited tumor xenograft growth in vivo, indicating that SLC14A1 downregulation may promote PCa progression by promoting cell growth. Based on the previous results, we validated the expression of cell-cycle related molecules and found that overexpression of SLC14A1 downregulated the transcription levels of several cell-cycle related genes (Fig. 4k). Further western blotting analysis showed that overexpression of SLC14A1 significantly reduced the protein expression of CDK1 and CCNB1 (Fig. 4l). In brief, these findings suggest that downregulation of SLC14A1 can promote cell growth and activate CDK1/CCNB1 cell cycle pathway.

### Overexpression of SLC14A1 inhibited cell metastatic ability and suppressed mTOR/MMP-9 signaling pathway

**Figure 5:**
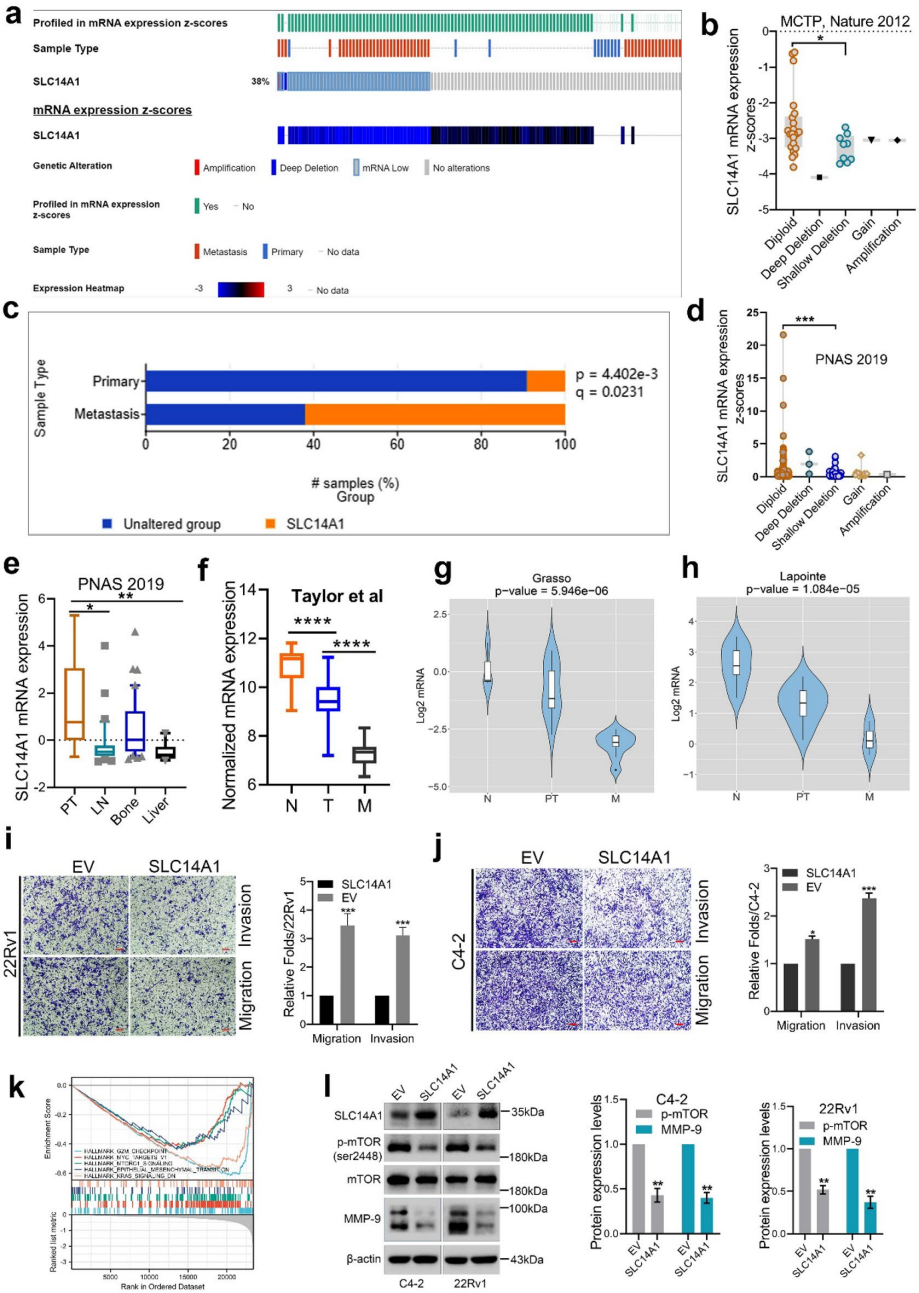
Overexpression of SLC14A1 inhibits cell metastatic ability and suppresses. mTOR/MMP-9 signaling pathway. (**a**) Genetic alterations of SLC14A1 gene in primary tissues and metastatic tissues of PCa based on public data from cbioportal database. (**b**) The correlation between the deletion of SLC14A1 gene and its mRNA expression in PCa from cbioportal. (**c**) Significant genomic alterations in the metastatic lesion of PCa compared to primary lesion from cbioportal database. (**d**) Shallow deletion of SLC14A1 in metastatic tissues of PCa leads to its low expression analyzed by public data from cbioportal. (**e**) SLC14A1 mRNA expression in metastases of PCa including lymph node, bone and liver analyzed from public data. (**f**–**h**) SLC14A1 mRNA expression in adjacent tissues, localized and metastatic tissues of PCa from GEO datasets including GSE21032 (Taylor et al.), GSE35988 (Grasso et al.) and GSE3933 (Lapointe et al.). (**i**–**j**) Representative Transwell data and quantification analysis of migration and invasion assays in C4-2 and 22RV1 cells transfected with lentivirus including SLC14A1 overexpression plasmid or empty vector (n = 3, mean ± SD). Scale bar = 100 μm. (**k**) Single-gene Gene Set Enrichment Analysis (GSEA) of SLC14A1 gene conducted by R language in TCGA. (l) Detection and analysis of protein expression level of mTOR, p-mTOR (ser2448) and MMP-9 in C4-2 and 22Rv1 cells transfected with lentivirus including SLC14A1 overexpression plasmid or empty vector by western blotting assay (n = 3, mean ± SD). *p < 0.05; **p < 0.01; ***p < 0.001; ****p < 0.0001. EV, empty vector.

We have mentioned that low expression of SLC14A1 may be associated with PCa metastasis in Fig. 1. First of all, we conducted a deep analysis of data from an article published on *Nature* in 2012 based on the cbioportal database and found that the SLC14A1 gene was significantly deleted in PCa tissues (Fig. 5a); In addition, it is worth noting that the degree of deletion in metastatic lesions is significantly higher than that in primary lesions (Fig. 5a,c), and its deletion can lead to low expression of SLC14A1 mRNA (Fig. 5b,d). Next, we further analyzed the expression of SLC14A1 in PCa metastatic tissues. We found that SLC14A1 is markedly downregulated in PCa metastatic tissues when compared with primary PCa tissues as evidenced by the analysis from public data published on *PNAS* in 2019 and GEO datasets including GSE21032 (Taylor et al.), GSE35988 (Grasso et al.) and GSE3933 (Lapointe et al.) (Fig. 5e–h). The above results indicate that SLC14A1 is significantly downregulated in metastatic lesions of PCa, and its low expression is likely caused by gene deletion.

To further verify that SLC14A1 is closely associated with cell migration and invasion, SLC14A1 was stably over-expressed in C4-2 and 22Rv1 cells by virus containing SLC14A1 plasmid. The ability of cells invasion and migration was detected by invasion and migration Transwell assays, as is shown in Fig. 5i–j, and we found that overexpression of SLC14A1 markedly decreased cell invasion and migration abilities. Next, to further explore the mechanisms by which SLC14A1 affects the metastasis of PCa cells, we conducted GSEA enrichment analysis of the SLC14A1 gene based on the TCGA database and R language. The results showed that downregulation of SLC14A1 significantly activated the mTOR signaling pathway, while also affecting the activity of the EMT signaling pathway (Fig. 5k). To verify this hypothesis, we conducted western blotting assay and found that overexpression of SLC14A1 significantly downregulated p-mTOR (ser2448) and MMP-9 levels (Fig. 5l). Taken together, these results to a large extent demonstrate that overexpression of SLC14A1 inhibits cell metastatic ability and suppresses the mTOR/MMP-9 signaling pathway.

## Single-cell transcriptomic analysis reveals that SLC14A1 expression is enriched in prostate basal-type cells

**Figure 6:**
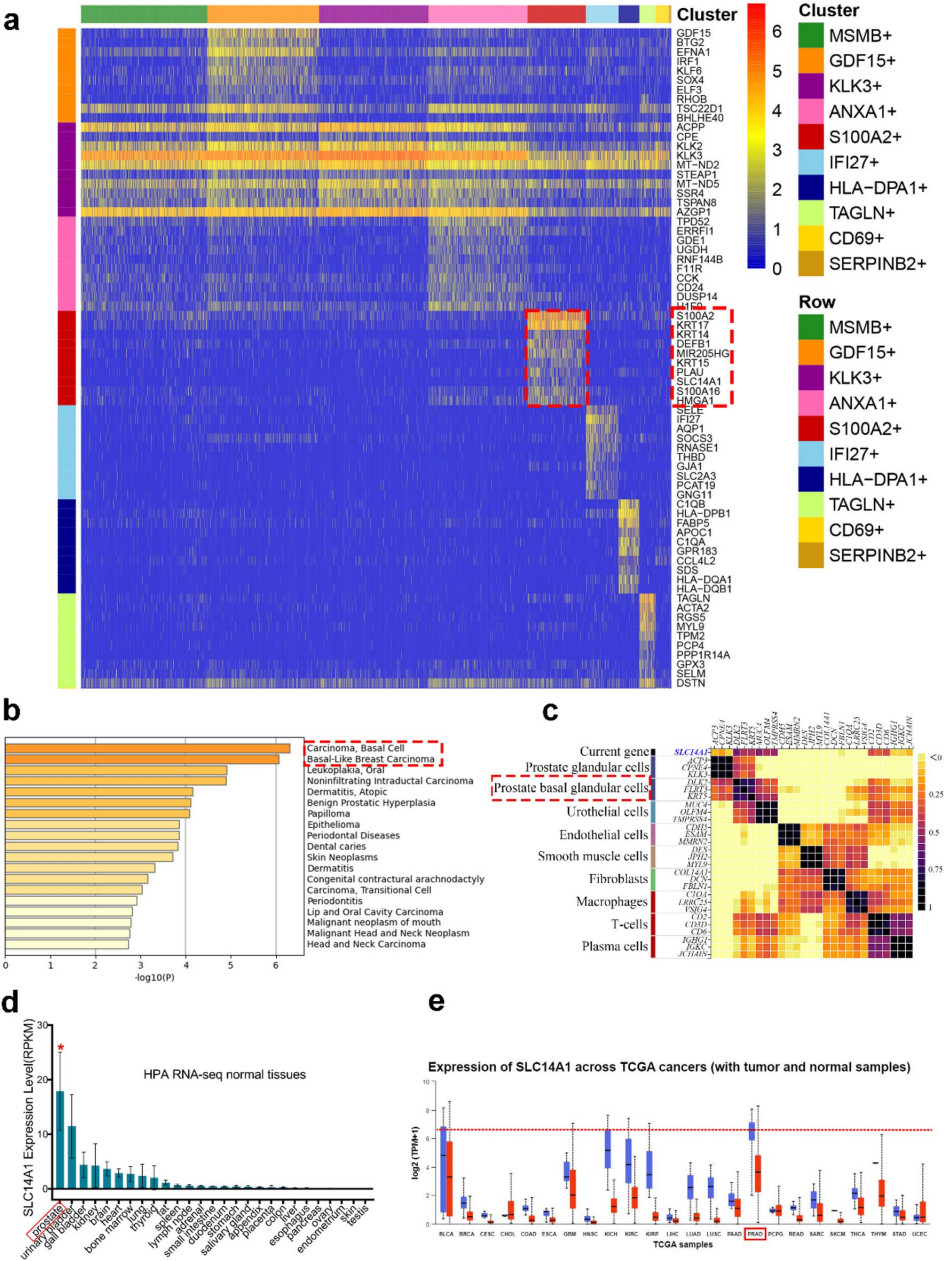
Single-cell transcriptomic analysis reveals that SLC14A1 expression is closely associated with prostate basal-type cells. (**a**) A heatmap of analysis of single-cell transcriptomic sequencing data of adult prostate in the GRNdb database (http://www.grndb.com/), which indicates SLC14A1 expression was mainly enriched in the SL00A2 + cluster. (**b**) GO enrichment analysis of the relative genes in the DisGeNET database (http://www.disgenet.org). (**c**) A heatmap shows analysis of the prostate single-cell transcriptome sequencing data from the THPA database (www.proteinatlas.org), which indicates SLC14A1 was mainly associated with prostate basal cells. (**d**) The transcription level of SLC14A1 in different normal tissues and organs of the human body. *only for annotation purpose. (**e**) mRNA expression of SLC14A1 across TCGA cancers with tumor and normal samples by the analysis from UALCAN website (https://ualcan.path.uab.edu/).

To investigate whether SLC14A1 could be a specific marker in prostate or its cancerous tissues, we firstly analyzed single-cell transcriptomic sequencing data of adult prostate in the GRNdb database^20^. As shown in Fig. 6a, SLC14A1 expression was mainly enriched in the SL00A2^+^ cluster, which consisted of S100A2, KRT17, KRT14, DEFB1, MIR205HG, KRT15, PLAU, SLC14A1, S100A16, HMGA1. We then performed GO enrichment analysis of the above genes in the DisGeNET database^21^ and found that these genes are closely associated with basal-type cells (Fig. 6b), indicating that SLC14A1 may be a potential marker of prostate basal cell. We further analyzed the prostate single-cell transcriptome sequencing data from the THPA database, and the results indicated that SLC14A1 was mainly associated with prostate basal cells (Fig. 6c). Notably, by analysis of the human tissue-specific expression by genome-wide integration of transcriptomics, we further analyzed RNA-seq data of normal tissues from BioProject (Accession: PRJEB4337) (Fig. 6d) and found that among all organ tissues in the human body, SLC14A1 has the highest transcription expression level in prostate tissues (Fig. 6e), suggesting the importance and specificity of SLC14A1 in prostate tissues. In conclusion, our results revealed that SLC14A1 can be used as a new marker of prostate basal cells.

## Materials and methods

### Data collection and processing

To identify the differential genes that indicate progression of PCa, we selected GSE3325 as the target of the study. Firstly, we downloaded the GSE3325 dataset based on the GEO database; Then, we obtained the differential genes downregulated in localized PCa (localized vs. normal, log FC < − 2 and adjusted *p* value < 0.05) and metastatic PCa (metastatic vs. localized, log FC < − 4 and adjusted *p* value < 0.05), respectively. We obtained the overlapping genes via the online Venn software called *Draw Venn Diagram* (http://bioinformatics.psb.ugent.be/webtools/Venn/), and finally SLC14A1 gene was selected as the study target.

## Survival Analysis

To analyze the relationship between SLC14A1 expression and PCa survival, we firstly analyzed the relationship between expression of SLC14A1 and progression-free survival (PFI) rate via applying R language. By further analyzing GSCA database (http://bioinfo.life.hust.edu.cn/GSCA/#/) we analyzed the relationship between expression of SLC14A1 and progression-free survival (PFS) rate or disease-free interval (DFI) rate. We further assessed whether a low SLC14A1 expression level leads to a poor disease-free survival (DFS) rate in PCa patients by analyzing GEPIA website (http://gepia2.cancer-pku.cn/#index) and GSE21034 dataset (Taylor et al.). The relationship between hypermethylation of cg26803305 and cg00377772 methylation sites in the promoter region of SLC14A1 gene with overall survival (OS) of PCa patients was analyzed by downloading and processing methylation dataset GSE46177.

### mRNA expression analysis

We downloaded data from the TCGA and GEO databases, including GSE6099 (Tomlins et al.) and GSE21032 (Taylor et al.) and analyzed the mRNA expression values of SLC14A1 in NAT, localized PCa tissues and metastatic PCa tissues. Meanwhile, we applied R 3.6.3 (ggplot2) and explored the expression of SLC14A1 in N stage and T stage based on TCGA database. Notably, we investigate the mRNA expression of SLC14A1 in cell lines on CCLE database (https://sites.broadinstitute.org/ccle/).

### Cell lines and reagents

We purchased the following human prostate or PCa cell lines: RWPE-1, P69, LNCaP, C4-2, C4-2B, 22Rv1 and DU145 from the American Type Culture Collection (ATCC, Manassas, VA). The LNCaP, C4-2, C4-2B, 22Rv1 and DU145 cell lines were cultured in RPMI 1640 medium supplemented with 10% fetal bovine serum (Gibco, NY) containing 1% of penicillin–streptomycin at 37 ℃ with 5% CO_2_. 2. The RWPE-1 and P69 cells were routinely cultured in keratinocyte-SFM in 5% CO_2_ at 37 ℃. MTT purchased from Sigma-Aldrich Co. (St. Louis, MO, USA) was gently dissolved in 5 mg/mL with PBS. pcDNA3.1-SLC14A1 plasmid was constructed by GenScript company (Nanjing, China), and the construct was validated by sequencing, and then the SLC14A1 gene was subcloned into pLenti CMV GFP Hygro (Addgene, MA, USA). To make the lentivirus, the pLenti CMV -SLC14A1, PAX2 and PMD2G plasmids were co-transfected into 293 T cells using X-tremeGENE HP DNATransfection Reagent (Roche, Switzerland) according to the manufacturer’s instructions. After 72 h transfection, the supernatants of 293 T cells were harvested and further used to transfect the PCa cells with the G418.

### RNA extraction and quantitative RT-PCR

In brief, total RNA of PCa cells was extracted with TRIzol reagent. 2 μL of extracted RNA was used for RNA quantification and was reverse transcribed using a Reverse Transcription Reaction Kit (purchased from TaKaRa PrimeScript™ RT Master Mix). Next, cDNA was amplified using specific primers. Corresponding primer sequences are listed in supplementary table S2. Notably, using 18S as an internal reference, relative changes in genes expression were normalized against 18S.

### Western blotting analysis

The procedure was described previously^22^. The following antibodies were used in this research: Antibodies against SLC14A1(#PA5-110377) was purchased from Thermo Fisher Scientific. Cyclin B1 (#12231), mTOR (#2983), p-mTOR (#5536) and β-actin (#3700) (Cell Signaling Technology, Beverly, MA, USA); MMP9 (ab38898) and CDK1 (ab133327) (Abcam, Cambridge, UK). All primary antibodies were diluted in 5% BSA at 1:1000.

### MTT assay

Briefly, relative PCa cells were plated into 96-well culture plates at the cell density of 5.0 × 10^3^/mL. After incubation for 24 h, 48 h and 72 h, the supernatant was changed with fresh RPMI 1640 medium containing 10% MTT (5 mg/mL) for another 4 h incubation. Next, the supernatant was removed and 150 µL DMSO was added into each well. The 96-well microplate reader (Bio-Rad, Hercules, USA) was used to detect the absorbance at the wavelength of 490 nm. Finally, we processed and visualized the data through graphpad9.0.

### Colony formation assay

After treatment with various desired conditions, the C4-2 and 22Rv1 cells in the logarithmic growth phase were gently seeded into each well of a 6-well plate (1000 cells/well) and further cultured with fresh RPMI 1640 supplemented with 10% FBS in an incubator at 37 °C with 5% CO_2_. About 1 week after seeding when colonic formation became visible, the medium was discarded and then the colonies were washed 2–3 times with PBS, then fixed with 4% paraformaldehyde for 15 min and further stained with crystal violet for 10 min. Then, the staining solution was gently washed away with running water. After the 6-well plates were allowed to dry naturally, the number of colonies was determined.

### Transwell assay

To investigate the migration and invasion abilities of PCa cells under given conditions (over-expression of SLC14A1 gene in C4-2 and 22Rv1 cells), migration and invasion assays were performed via Boyden chambers with an 8-μm pore size (Millipore, Germany). For the migration assay, chambers plated into 24-well plates were seeded with 4 × 10^4^ C4-2 or 3 × 10^4^ 22Rv1 cells suspended in 200 μL serum-free 1640 medium in the upper chamber without Matrigel, and 800 μL 1640 medium with 10% FBS was added to the lower chamber for 24 h. For the invasion assay, 60 μL Matrigel (Sigma, St. Louis, MO, USA) was added to the upper chamber and incubated in a cell incubator at 37 °C for 4 h, then 8 × 10^4^ C4-2 or 6 × 10^4^ 22Rv1 cells suspended in 200 μL serum-free culture 1640 medium were added to the upper chamber and 800 μL 1640 medium containing 10% FBS was added to the lower chamber for 48 h. After washing with PBS 3 times, fixing with 4% paraformaldehyde for 15 min and further staining with 0.1% crystal violet for 5 min, the visible cells were observed and counted under an inverted light microscope (magnification, × 100) in five random fields for each chamber.

### DNA methylation analysis

To investigate the methylation status of SLC14A1 promoter, we analyzed the GSCA database (mainly based on TCGA) (http://bioinfo.life.hust.edu.cn/GSCA/#/). Meanwhile, we analyzed the methylation status of SLC14A1 promoter in PCa progression from GEO datasets including GSE112047, GSE157272 and GSE46177.

### Tissue chip and immunohistochemistry (IHC) assays

Notably, the immunohistochemical results we presented were obtained from the THPA database (www.proteinatlas.org). Briefly, the Human Protein Atlas is a Swedish-based program initiated in 2003 with the aim to map all the human proteins in cells, tissues, and organs using an integration of various omics technologies, including antibody-based imaging, mass spectrometry-based proteomics, transcriptomics, and systems biology^23^. The protein expression data from 44 normal human tissue types is derived from antibody-based protein profiling using conventional and multiplex immunohistochemistry. All underlying images of immunohistochemistry stained normal tissues are available together with knowledge-based annotation of protein expression levels^23^.

### Single-cell transcriptomic analysis

To further explore the important role of SLC14A1 in prostate or PCa, we analyzed the single-cell transcriptomic data of adult prostate through GRNdb database (http://www.grndb.com/). We further analyzed the prostate single-cell transcriptome sequencing data from the THPA database (www.proteinatlas.org).

### Functional enrichment analysis

According to the median score of SLC14A1 expression, PCa patients in TCGA database were divided into high and low SLC14A1 expression groups. The R 3.6.3 DESeq2 was used to perform the DEGs analysis between these two groups. Then, DEGs were visualized as volcano plots by R3.6.3 ggplot2. Notably, the correlation between the expression of the cell cycle related genes and SLC14A1 was assessed by using Spearman’s correlation analysis. Functional enrichment analyses including GO and KEGG analysis were implemented for the DEGs using the R3.6.3. Gene set enrichment analysis (GSEA) was carried out using the R 3.6.3 with clusterProfiler.

### PCa xenograft animal model

All animal experiments were approved by the Ethical Committee of the First Affiliated Hospital of Medical College, Xi’an Jiaotong University, Xi’an, China. And care for them was in accordance with guidelines of Institutional Animal Care and Use Committee of Xi’an Jiaotong University. Twenty 4-week-old BALB/c male nude mice purchased from the Experimental Animal Center of Xi’an Jiaotong University were randomly separated into two experimental groups with each group containing 10 nude mice and then injected subcutaneously in both flanks with 100 μL serum-free 1640 culture medium containing 50 μL Matrigel (Sigma-Aldrich; Merck KGaA) and 5 × 10^6^ 22Rv1 cells (empty vector or SLC14A1-overexpressed) after 7 days of feeding. We observed the growth and tumorigenesis of the mice on a daily basis. 18 days after inoculation, tumor size was measured by caliper and recorded every two days until the mice with tumors were euthanized by the cervical dislocation method under anesthesia using 0.7% sodium pentobarbital to harvest the tumors at day 30. The tumor volume was calculated as follows: volume (mm3) = 1/2 × length × width^2^. Notably, at the end of the nude mice experiment, all tumors were excised, weighed, and then fixed in 4% paraformaldehyde for further analysis. In this study, the humane endpoints for euthanasia mainly two points: 1) The tumor should not exceed 10% of the animal's normal body weight; 2) A tumor should not exceed 20 mm in any one dimension.

### Statistical analysis

Briefly, GraphPad Prism version 9.0 software (GraphPad, SanDiego, CA, USA) was applied for analyzing differences between two groups (Student’s t test). *P* < 0.05 was regarded as the threshold value for statistical significance. All error bars in graphical data represent mean ± SD.

### Ethical statement

All protocols used for animal manipulation were approved by the Institutional Animal Care Committee of Xi’an Jiao Tong University, and all efforts were made to minimize the pain of animals and reduce the number of animals used in experiments. All methods are reported in accordance with ARRIVE guidelines.

## Discussion

There are various factors that can promote the occurrence and development of PCa, however, we still need to further explore the potential mechanisms and markers of PCa development. In this study, we initially performed data analysis on PCa progression through mining GEO dataset and identified that the mRNA expression of SLC14A1 is frequently and gradually decreased during PCa progression. Furthermore, we validated this finding in PCa specimens by mining on the TCGA portal. Furthermore, through analysis of the human tissue-specific expression by genome-wide integration of transcriptomics from BioProject, we found that among all organ tissues in the human body, SLC14A1 has the highest transcription expression level in prostate tissues, suggesting the importance and specificity of SLC14A1 in prostate tissues. In our results, SLC14A1 expression was mainly enriched in SL00A2^+^ cluster, which consists of genes indicating prostate basal cells, indicating that SLC14A1 may be a potential marker of prostate basal cell, which is beneficial for us to better understand the molecular properties of prostate basal cell. However, what's more important is that we further demonstrated that SLC14A1 not only was viewed as a marker of prostate basal cells, but also it plays an important role in the progression of PCa, such as cell growth and metastasis.

In this study, our results demonstrated that SLC14A1 is significantly downregulated in PCa progression, however, the potential mechanisms of its low expression are still unclear. Thus, we firstly analyzed from two aspects: epigenetics and genomic instability, which are the most common mechanisms of transcriptional down-regulation of gene expression in PCa. Recent studies have demonstrated that downregulation of SLC14A1 in Urothelial carcinomas (UCs) is attributed to epigenetic silencing^13^. Based on this finding, we further analyzed the public data and found that the methylation level of the CpGs island of SLC14A1 promoter region in PCa tissues and cancer cells is significantly increased compared to NAT, and mRNA expression of SLC14A1 is negatively related to its methylation level. Meanwhile, we demonstrated that hypermethylation on SLC14A1 promoter to a large extent is associated with progression of PCa by analyzing the methylation level of SLC14A1 promoter in PCa tissues with different Gleason score and ki-67. In addition, hypermethylation of SLC14A1 promoter is closely related to poor prognosis of PCa patients. Therefore, epigenetic silencing (hypermethylation) is a critical mechanism for the low expression of SLC14A1. Nevertheless, genomic instability, such as deletion, can also lead to gene low expression. In this study, high frequency deletion was detected in the coding DNA sequence of the SLC14A1 gene while rare somatic mutation occurred, implying deletion is another potential mechanism contributing downregulation of SLC14A1. Notably, deletion of SLC14A1 also occurs in PCa metastases, implying the deletion of SLC14A1 gene runs through the development and progression of PCa. Transcriptional downregulation of many genes in PCa is limited to one of two mechanisms: methylation or deletion. For example, the transcriptional downregulation of *PTEN* and *GSTP1* is attributed to genomic deletion and promoter methylation, respectively. Therefore, the mechanisms of down-regulation of SLC14A1 indicate that the downregulation of a gene can be caused by many mechanisms at the same time.

To identify the function of SLC14A1 in PCa, by GO, KEGG and GSEA enrichment analysis in TCGA database, we found that downregulation of SLC14A1 activates cell cycle pathway and MTORC1 signaling pathway. Among cell cycle related genes, CDK1 and CCNB1 had the most significant changes verified by bioinformatics and experiments. In vitro, we experimentally found that SLC14A1 overexpression downregulated p-mTOR (S2448), which is an active component of mTORC1 pathway^24^. mTOR is a classic protein kinase that controls cellular survival, proliferation, migration, metabolism, catabolism, immune responses and autophagy to maintain cellular homeostasis^25^. Amplification, mutations, and overexpression in the upstream genes, including oncogenes and tumor suppressor genes result in the activation of the mTOR signaling cascade in human malignancies^26^, such as *AKT, PIK3CA, KRAS, IGFR*, and *EGFR*^25^*.*Among them, the PI3K/AKT/mTOR pathway is one of the most important growth regulatory pathways in cancers^27^. In urothelial carcinoma, it has been identified that SLC14A1 downregulated p-mTOR(S2448)^13^. Thus, as shown in Figs. 4 and 5, CDK1/CCNB1 and MMP-9 probably be downstream factors of p-mTOR pathway, which contribute to proliferation or metastasis of PCa cells. However, the mechanism of how the mTOR signaling pathway is activated after SLC14A1 downregulation is still not clear and under investigation. We assume that SLC14A1 downregulation may lead to accumulation of urea and arginine, and high concertation of arginine may activate the nutrient sensing kinase mTOR. This hypothesis will be test in the future.

Recently, it has reported that upregulation of SLC14A1 expression levels inhibited the proliferation, invasion, and metastatic ability of renal cancer cells^28^. It also has been demonstrated that the low expression level of SLC14A1 closely correlates with the occurrence, invasion and progression of bladder urothelial carcinoma^29,30^. More importantly, bladder cancer (BC) patients with high proportions of intratumoral SLC14A1^+^ CAFs show cancer stage-independent poor outcome and a worse response rate to neoadjuvant chemotherapy or immunotherapy^16^. In PCa, SLC14A1 is a novel important gene associated with biochemical recurrence (BCR)^17^. Based on these findings, we analyzed the correlation between low expression of SLC14A1 and prognosis of PCa patients in the TCGA database and found that the lower expression level of SLC14A1 was correlated to the lower survival rate (including PFI, PFS, DFS and DFI) of patients. TNM stage is the most important risk factor for PCa patients’ long-term prognosis, herein, by analysis of TCGA data, we found that lower SLC14A1 level is closely related to higher T stage or N stage. Therefore, low expression of SLC14A1 is closely related to poor prognosis or progression in PCa patients, suggesting that its low expression could promote the progression of PCa. In this study, based on bioinformatics analysis and experiments, we found overexpression of SLC14A1 inhibited cell proliferation and growth, and decreased cell migration and invasion abilities. Similarly, in non-small cell lung cancer, it has been demonstrated that SLC14A1 inhibited the proliferation and migration of NSCLC cell lines^31^. In addition, Li et al. found that overexpression of SLC14A1 in mouse melanoma cell line B16 decreased cell viability and proliferation, and increased apoptosis^32^. Overall, SLC14A1 deficiency is detected in various types of tumors, and its low expression is closely linked to tumor progression (mainly including proliferation, invasion and migration).

In conclusion, our results indicate that downregulation of SLC14A1 is closely associated with PCa progression and metastasis, and that downregulation activated the mTOR signaling pathway. These findings suggest that SLC14A1 plays an important role in the regulation of growth and metastasis of PCa, and that downregulation of SLC14A1 and the hypermethylation on its promoter could serve as potential markers for PCa prognosis and progression.

### Supplementary Information

Supplementary Information 1.

Supplementary Information 2.

## Data Availability

The datasets analyzed during the current study are available in the GEO database repository, including GSE3325, GSE112047, GSE157272, GSE46177, GSE21034, GSE21032 and GSE6099. We can enter the above ID numbers in the following website to get the raw data: Home - GEO DataSets - NCBI (nih.gov).
