# Supplementary material for: Down-regulation of SLC14A1 in prostate cancer activates CDK1/CCNB1 and mTOR pathways and promotes tumor progression
Source: Sci Rep. 2024 Jun 28;14:14914. doi: 10.1038/s41598-024-66020-1 (PMC11213927; doi:10.1038/s41598-024-66020-1)
Supplement: Supplementary file 2 — Supplementary Information 2. [file 41598_2024_66020_MOESM2_ESM.docx]

**Fig. S1 Low expression of SLC14A1 may also be related to its gene deletion**


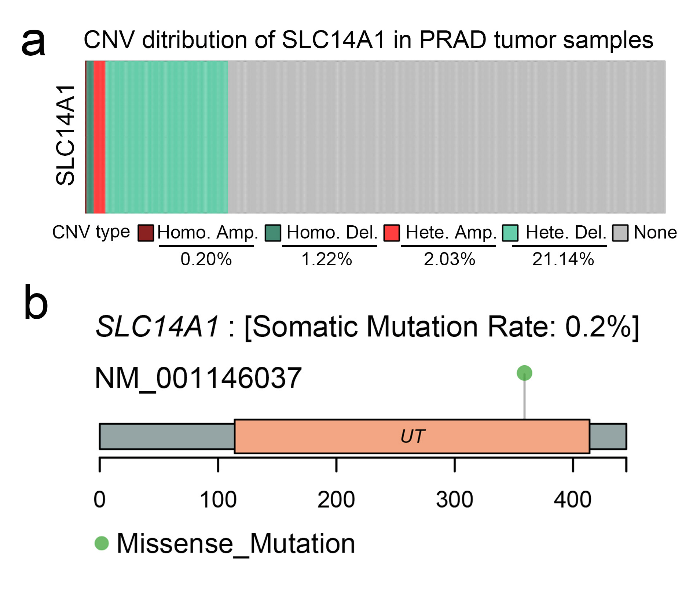


**Fig. S1** Low expression of SLC14A1 may also be related to its gene deletion. **(a)** Analysis of SLC14A1 gene copy-number alterations in PCa from GSCA website based on TCGA. **(b)** Analysis of SLC14A1 gene somatic mutation rate in PCa from GSCA database.

**Fig. S2** **DNA methyltransferase DNMT3B may mediate methylation of the SLC14A1 promoter region and lead to its low expression**


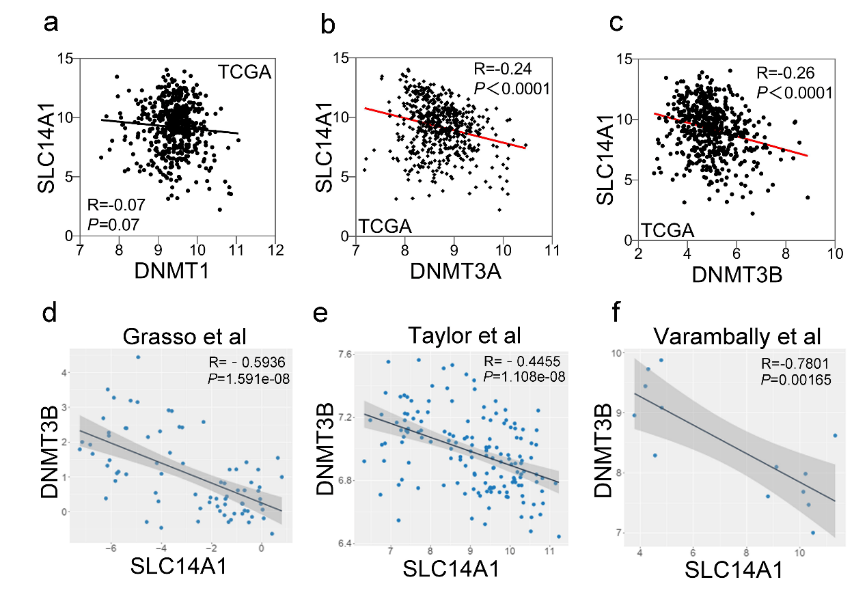


**Fig. S2** DNA methyltransferase DNMT3B may mediate methylation of the SLC14A1 promoter region and lead to its low expression. **(a-c)** Analysis of linear correlation between SLC14A1 and DNMT1, DNMT3A or DNMT3B from TCGA. **(d-f)** Analysis of linear correlation between SLC14A1 and DNMT3B from GEO datasets including GSE35988 (Grasso et al), GSE21032 (Taylor et al) and GSE3325 (Varambally et al).


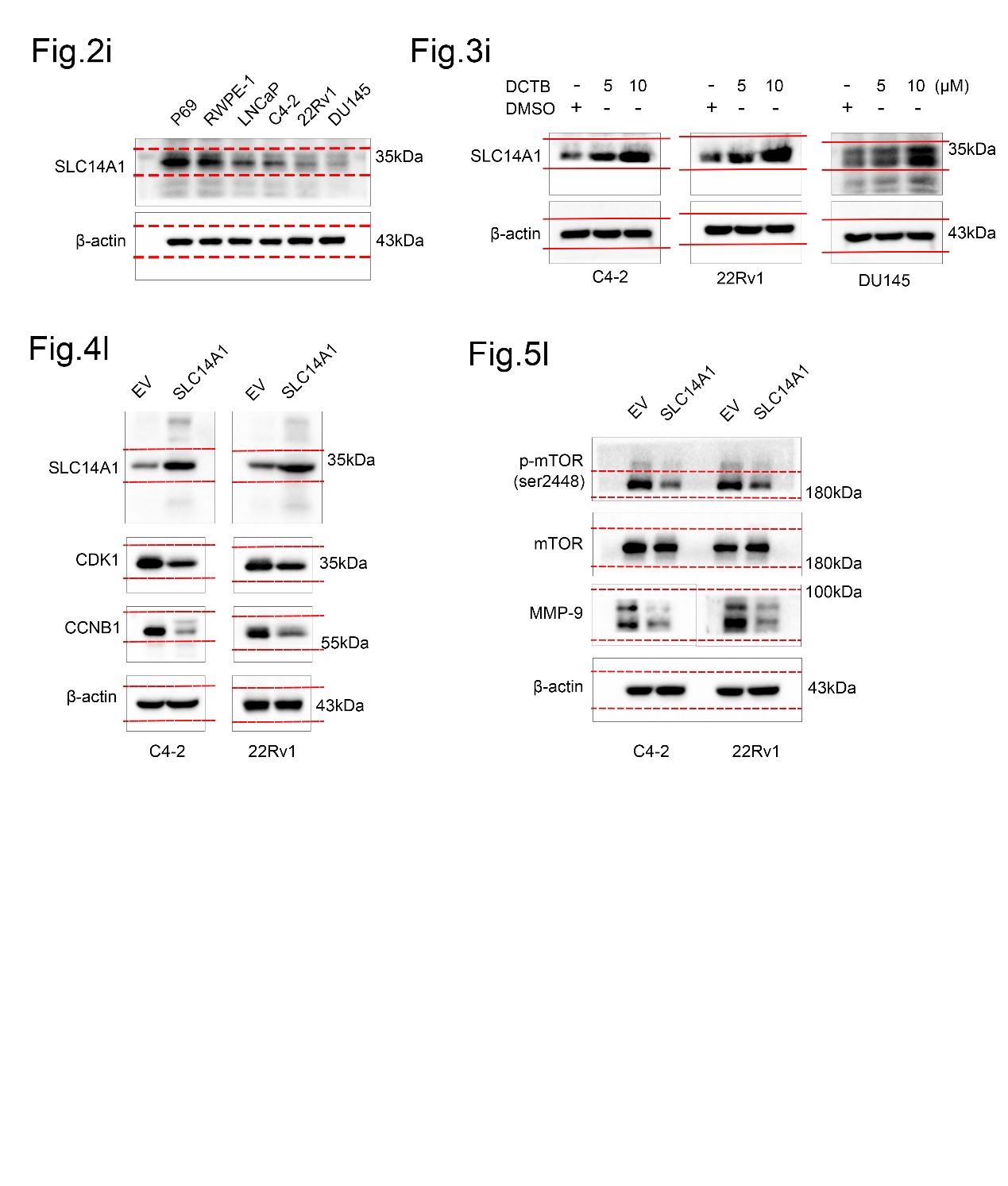


The above is the raw data for the results of western blots in the article, the red line, whether dashed or realized, represents the part that is cropped. Notably, in this study, based on the purpose and needs of the experiment, we firstly cut the whole membrane of blots into strips and then incubated the antibodies, that is, we did not incubate the antibodies on the entire membrane of blots. **Each indicator is repeated three times, and all the duplicate blots are on the next page.**

Fig. 2i the grouping of gels/blots (SLC14A1 and β-actin) cropped from different gels.

Fig. 3i the grouping of gels/blots (SLC14A1 and β-actin) cropped from different gels.

Fig. 4l the grouping of gels/blots (SLC14A1, CDK1, CCNB1 and β-actin) cropped from different gels.

Fig. 5l the grouping of gels/blots (p-mTOR and β-actin) cropped from different parts of the same gel; The grouping of gels/blots (p-mTOR, mTOR and MMP-9) cropped from different gels.


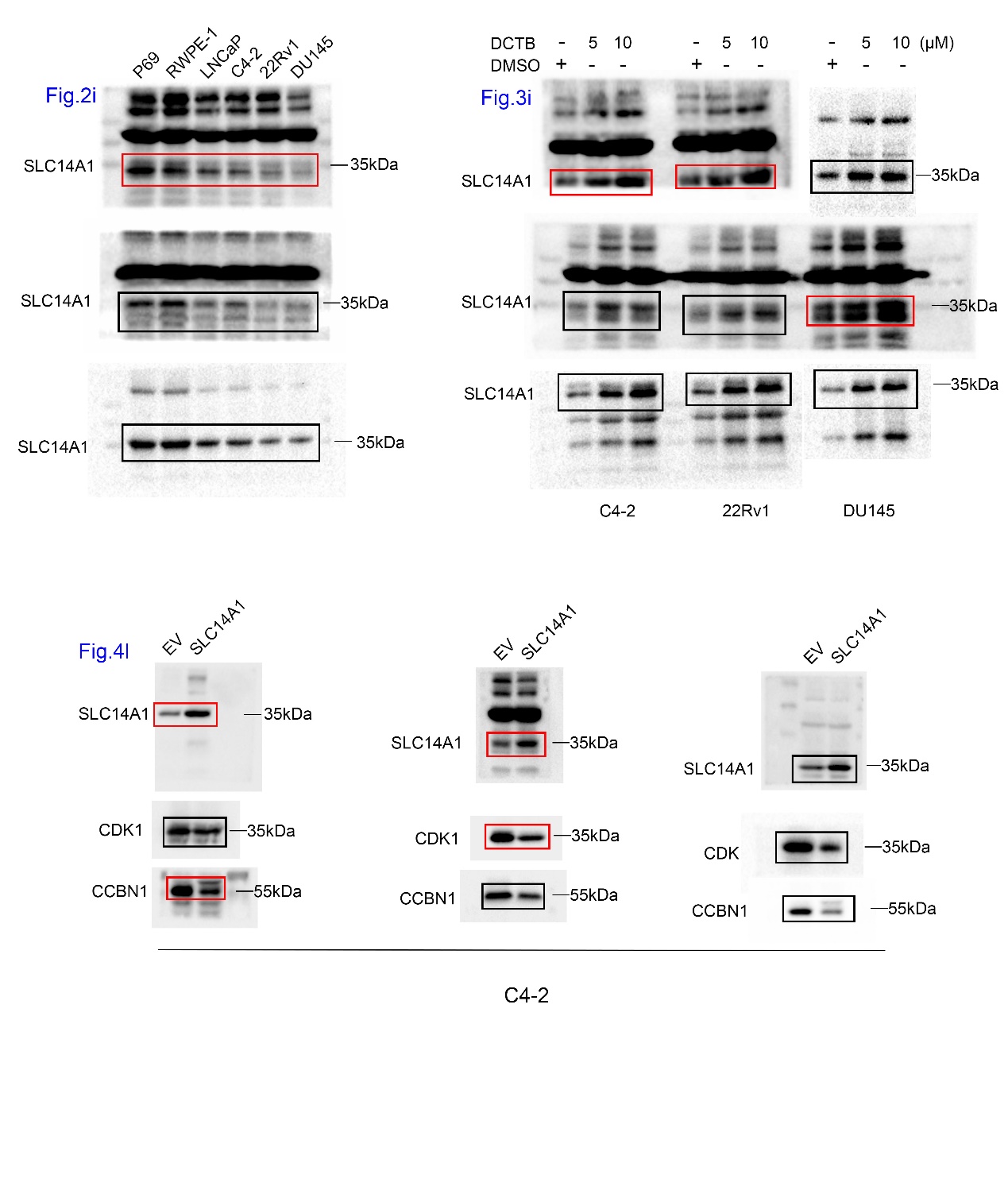


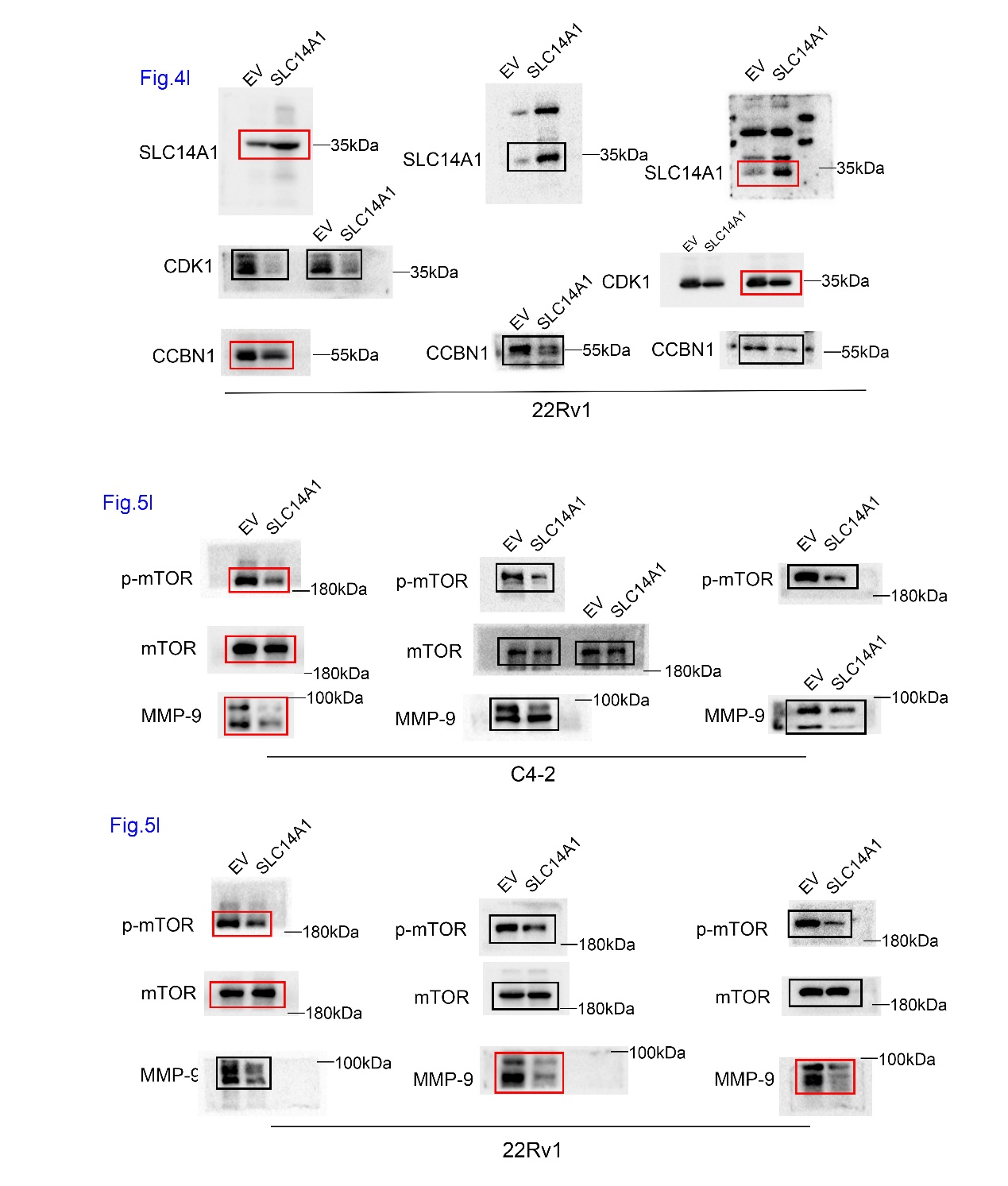


1. Original images of full-length blots for all replicates were provided above.
2. It is worth noting that **the blots were cut prior to hybridisation with antibodies during blotting**. The above blots are original and have not been cropped in any way.
3. The red box indicates the blots placed in the body of the text and the corresponding crop position. The black box indicates duplicate blots and the corresponding crop position.

Table S1 Number of patients that have high and low levels of SLC14A1 with different Characteristics

| Characteristic | Low expression of SLC14A1 | High expression of SLC14A1 | p |
| --- | --- | --- | --- |
| n | 249 | 250 |  |
| T stage, n (%) |  |  | **0.030** |
| T2 | 81 (16.5%) | 108 (22%) |  |
| T3 | 159 (32.3%) | 133 (27%) |  |
| T4 | 7 (1.4%) | 4 (0.8%) |  |
| N stage, n (%) |  |  | **0.008** |
| N0 | 164 (38.5%) | 183 (43%) |  |
| N1 | 51 (12%) | 28 (6.6%) |  |
| M stage, n (%) |  |  | 1.000 |
| M0 | 230 (50.2%) | 225 (49.1%) |  |
| M1 | 2 (0.4%) | 1 (0.2%) |  |
| Gleason score, n (%) |  |  | **< 0.001** |
| 6 | 18 (3.6%) | 28 (5.6%) |  |
| 7 | 106 (21.2%) | 141 (28.3%) |  |
| 8 | 33 (6.6%) | 31 (6.2%) |  |
| 9 | 88 (17.6%) | 50 (10%) |  |
| 10 | 4 (0.8%) | 0 (0%) |  |
| OS event, n (%) |  |  | 0.544 |
| Alive | 243 (48.7%) | 246 (49.3%) |  |
| Dead | 6 (1.2%) | 4 (0.8%) |  |
| PFI event, n (%) |  |  | **0.002** |
| Alive | 188 (37.7%) | 217 (43.5%) |  |
| Dead | 61 (12.2%) | 33 (6.6%) |  |

| SLC14A1 | F: 5ʹ-ACTATGGTTAGAGTGGACAGCC-3ʹ; |
| --- | --- |
|  | R: 5ʹ-ACGGGTTTGTCTTTAAGCTGG-3ʹ. |
| 18S | F: 5ʹ-GCAATTATTCCCCATGAACG-3ʹ; |
|  | R: 5ʹ-GGCCTCACTAAACCATCCAA-3ʹ. |
| CDK1 | F: 5ʹ- GGATGTGCTTATGCAGGATTCC-3ʹ; |
|  | R: 5ʹ- CATGTACTGACCAGGAGGGATAG-3ʹ. |
| CDK2 | F: 5ʹ- CCAGGAGTTACTTCTATGCCTGA-3ʹ; |
|  | R: 5ʹ- TTCATCCAGGGGAGGTACAAC-3ʹ. |
| CDK3 | F: 5ʹ- CCAGCTCTTTCGTATCTTTCGT-3ʹ; |
|  | R: 5ʹ- TTCCTGGTCCACTTAGGGAAG-3ʹ. |
| CDK4 | F: 5ʹ- ATGGCTACCTCTCGATATGAGC-3ʹ; |
|  | R: 5ʹ- CATTGGGGACTCTCACACTCT-3ʹ. |
| MYC | F: 5ʹ- GTCAAGAGGCGAACACACAAC-3ʹ; |
|  | R: 5ʹ- TTGGACGGACAGGATGTATGC-3ʹ. |
| CCNA2 | F: 5ʹ- GGATGGTAGTTTTGAGTCACCAC-3ʹ; |
|  | R: 5ʹ- CACGAGGATAGCTCTCATACTGT-3ʹ. |
| CCNB1 | F: 5ʹ- AATAAGGCGAAGATCAACATGGC-3ʹ; |
|  | R: 5ʹ- TTTGTTACCAATGTCCCCAAGAG-3ʹ. |
| CCNB2 | F: 5ʹ- TGCTCTGCAAAATCGAGGACA-3ʹ; |
|  | R: 5ʹ- GCCAATCCACTAGGATGGCA-3ʹ. |
| CCNB3 | F: 5ʹ- ATGAAGGCAGTATGCAAGAAGG-3ʹ; |
|  | R: 5ʹ- CATCCACACGAGGTGAGTTGT-3ʹ. |
| CCNE1 | F: 5ʹ- GCCAGCCTTGGGACAATAATG-3ʹ; |
|  | R: 5ʹ- CTTGCACGTTGAGTTTGGGT-3ʹ. |
| CCNE2 | F: 5ʹ- TCAAGACGAAGTAGCCGTTTAC-3ʹ; |
|  | R: 5ʹ- TGACATCCTGGGTAGTTTTCCTC-3ʹ. |

Table S2 Primer information
